# Integrated Curcumin-Based Polylactic Acid Film with Screen-Printed Indicator for Real-Time Shrimp Freshness Monitoring

**DOI:** 10.3390/foods15081453

**Published:** 2026-04-21

**Authors:** Kelan Liu, Shasha Zhang, Xiaoxue Han, Yuye Zhong, Shaoyun Huang, Xianwen Ke

**Affiliations:** 1 Electronic Information School, Wuhan University, Wuhan 430072, China; 2023202170005@whu.edu.cn (K.L.); zhang_ss@whu.edu.cn (S.Z.); 2023102170002@whu.edu.cn (X.H.); 2013301750088@whu.edu.cn (Y.Z.); 2 School of New Energy, Jingchu University of Technology, Jingmen 448000, China; huangshaoyun@jcut.edu.cn

**Keywords:** intelligent packaging, food freshness monitoring, curcumin, polylactic acid

## Abstract

To reduce food waste and mitigate health risks from accidentally consuming spoiled food, freshness-indicating technologies are increasingly demanded. However, conventional colorimetric-based freshness-indicating packaging is limited by instability, subtle color changes, and complex production processes. This study presents a curcumin-based ink suitable for eco-friendly polylactic acid (PLA) food packaging films enabling real-time shrimp freshness monitoring via integrated intelligent packaging. The ink comprised curcumin as the indicator, ethyl cellulose (EC) and polyvinyl butyral (PVB) as binders, and polyethylene glycol 400 (PEG 400) to regulate permeability. Excellent printability was demonstrated by fineness, initial dryness and fluidity tests. It also demonstrated good thixotropic, viscosity, and flow curve properties. Printing minimally affected the PLA films’ mechanical and barrier properties. The indicator label showed high sensitivity, rapid response, and excellent reversibility to ammonia vapor. Practical application in monitoring shrimp spoilage at 25 °C and 4 °C revealed a strong correlation between the distinct color transition of the label and the increase in total volatile basic nitrogen (TVB-N) content and pH value, providing a reliable visual warning before obvious spoilage signs appeared. This work provides a viable integrated indicator packaging strategy for developing intelligent packaging, offering significant potential to reduce food waste and enhance supply chain transparency for perishable goods.

## 1. Introduction

Rising living standards and health awareness have intensified consumer demand for high-quality food, particularly in terms of freshness [1]. Freshness is critical not only for sensory and nutritional quality but also for food safety [2]. Conventional packaging, however, provides no real-time information on food status [3], and consumers frequently misjudge freshness [4]. These limitations contribute directly to two major issues: substantial food waste [5] and public health risks from foodborne illnesses [6]. Consequently, intelligent packaging that enables real-time monitoring and visual food quality indication has become an important research focus in food packaging.

**Figure 1:**
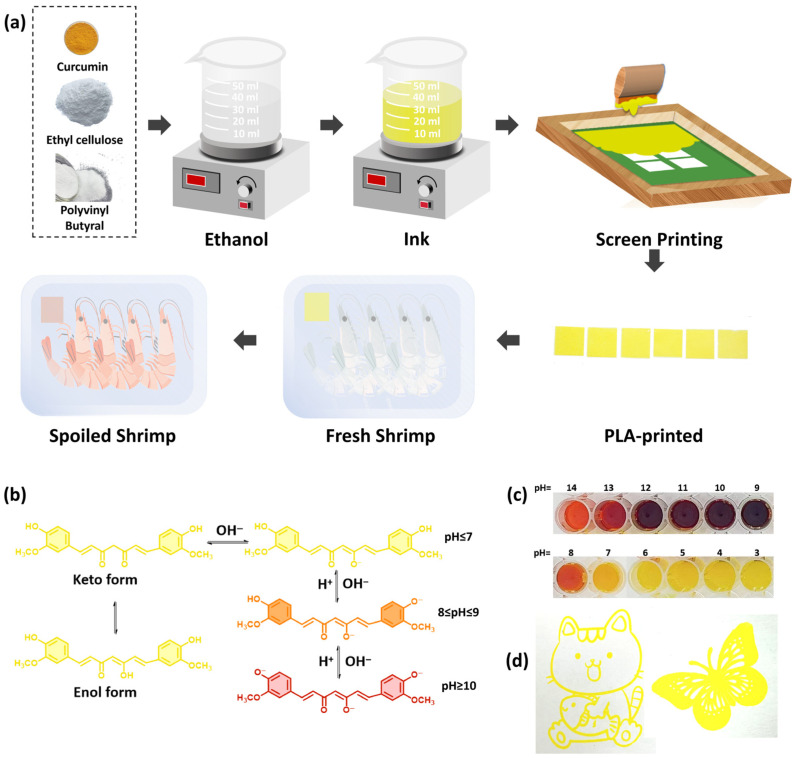
Schematic illustration of the curcumin-based freshness-indicating ink: (**a**) Preparation, screen printing, and application. (**b**) Color-changing mechanism of curcumin. (**c**) Color response of the indicator ink at different pH values. (**d**) Outline pattern printing effect on PLA film.

Among various intelligent packaging technologies, colorimetric freshness indicators are especially promising due to their low cost, intuitive response, and operational simplicity [7,8]. These systems integrate indicators that convert internal packaging conditions (e.g., pH, humidity, specific gases, and metabolites) into visible color changes, providing real-time freshness information [9]. For protein-rich foods like pork and shrimp, spoilage produces volatile basic nitrogen compounds (TVB-N), which can be indicated by pH-responsive materials [10,11]. Various natural pH-sensitive pigments, including anthocyanins (red to purple to cyan), shikonin (red to purple), betalains (purplish-red to yellow), and curcumin (yellow to reddish-brown), have been extensively explored for freshness-indicating applications [12,13,14,15,16]. Among these, curcumin exhibits noticeable color transitions via keto-enol tautomerism in response to pH changes. Under neutral or acidic conditions, curcumin presents a yellow color in the enol form, while it turns reddish-brown in the deprotonated enolate form under alkaline conditions (Figure 1b) [17,18,19]. It also offers significant non-toxicity, antioxidant and antibacterial properties, as well as compatibility with various polymer matrices. These advantages make curcumin a particularly attractive candidate for integration into intelligent packaging systems [20,21,22].

The stable integration of functional indicators into food packaging while maintaining their consistent and durable responsiveness under practical conditions remains a critical challenge. Current methods, such as solution casting or adhesive labeling, often lack robustness or integrability [23,24]. Among various intelligent food packaging strategies, printed indicator labels have attracted considerable attention owing to their good flexibility, ease of integration with commercial packaging lines, and suitability for large-scale production. Jitjamrasrat et al. [25] prepared a dual-mode fluorescent indicator label for real-time shrimp freshness detection by responding to spoilage-induced volatile amines. Luo et al. [26] fabricated gradient colorimetric indicators via inkjet printing to sense pH and amine changes for fish freshness evaluation. Screen printing has recently emerged as a promising alternative for fabricating integrated indicator packaging, offering advantages in patternability, cost, scalability, and compatibility with flexible substrates [27,28,29,30,31]. In this technique, the choice of binder is crucial. EC offers excellent film-forming ability and compatibility with biodegradable PLA [32], while PVB provides strong adhesion, flexibility, and durability to printed patterns [33].

Although various indicator systems and printing techniques have been reported in recent studies, most of them focus only on color response performance, while ignoring the printability on biodegradable polymer films. This work presents a novel integrated intelligent packaging system by screen-printing a curcumin-based indicator ink onto PLA film, using EC/PVB composite binders and PEG 400 as a modifier. The ink exhibited excellent printability and stability, producing clear and aesthetically pleasing patterns through screen printing. The printed indicator labels exhibited uniform color distribution, clear boundary definition, and high stability during storage and application. Meanwhile, the mechanical properties and barrier performance of the printed PLA films were minimally affected. Finally, its practical application for real-time freshness monitoring of shrimp was successfully demonstrated. This study provides a feasible strategy for constructing high-performance printed intelligent packaging for food applications.

## 2. Materials and Methods

### 2.1. Materials

Curcumin, EC (ethoxyl content: 48–49.5%, viscosity: 200 mPa·s), PVB (Mw: 25,000–40,000), and PEG 400 were purchased from Mackin Biochemical Co., Ltd. (Shanghai, China). Absolute ethanol, acetic acid, ammonia solution and hydrochloric acid were purchased from China National Pharmaceutical Group Co., Ltd. (Shanghai, China). Boric acid, magnesium oxide, methyl red, and bromocresol green were obtained from Aladdin Biochemical Technology Co., Ltd. (Shanghai, China). Distilled water was prepared using a Milli-Q system (UPW-I-5 T, Sichuan ULUPURE Ultrapure Technology Co., Ltd., Chengdu, China). Polylactic acid food preservation film (thickness: 12 μm) was supplied by Changzhou Bojiang New Materials Technology Co., Ltd. (Changzhou, China) Fresh shrimps were procured from a local market in Wuhan, China.

### 2.2. Preparation of Curcumin-Based Freshness-Indicating Ink

EC and PVB, with a total mass of 1 g, were dissolved in 14 mL of ethanol. The mixture was stirred overnight at 200 rpm using a magnetic stirrer at room temperature to obtain a transparent ink. Referring to the literature [34,35] and preliminary experiments, 0.02 g of curcumin powder was added, and stirring was continued for 4 h. Then, 0.05 g of PEG 400 was added and the mixture was stirred for 4 h to obtain the curcumin-based freshness-indicating ink (Figure 1). The prepared ink was degassed in a vacuum drying oven to remove air bubbles. Following Table 1, inks with different mass ratios of EC and PVB were designated as Ink-1, Ink-2, Ink-3, Ink-4, Ink-5, and Ink-6. The pH value of the inks was measured to be approximately 7.5, a weakly alkaline state that ensures the ink remains stably yellow at the initial stage of packaging.

### 2.3. Characterization and Printability Properties of Inks

#### 2.3.1. Particle Size and Zeta Potential of Inks

The sample was diluted with absolute ethanol to approximately 0.1 wt%. The particle size distribution and zeta potential of the sample were measured using a Nano-ZS nanoparticle size and zeta potential analyzer (Malvern Instruments, Malvern, UK).

#### 2.3.2. Initial Dryness of Inks

The initial dryness of the ink was tested according to the method specified in GB/T 13217.5-2023 [36] for liquid inks. The ink was placed at the 100 μm mark on the scraper plate. The blade was used to scrape down vertically quickly. After 30 s, a test paper was aligned with the 0 μm mark and laid flat. A rubber roller was used to roll evenly from the 0 μm to the 100 μm mark. The test paper was then peeled off. The length of the ink trail was measured, and the initial drying performance of the ink was recorded.

#### 2.3.3. Fluidity of Inks

The fluidity of the ink was measured according to GB/T 14624.3-2008 [37]. 0.1 mL of ink was pipetted and placed at the center of the circular glass plate in the fluidity tester. Another circular glass plate was then placed on top. Once the ink overlapped between the two plates, a 100 g weight was applied for 15 min. After the time elapsed, the weight was removed, and the diameter of the spread ink was measured. The measurement was considered valid only if the difference between the maximum and minimum diameters was less than 2 mm.

#### 2.3.4. Fineness of Inks

The fineness of the ink was tested according to the particle fineness method specified in GB/T 13217.3-2022 [38]. The ink was placed into the groove of a scraper fineness tester. A scraper was drawn down vertically and evenly with force within 3 s until reaching the 0-micron scale line. The ink film surface was then observed at a 15–20° angle. The particle density value was read on the ink film surface within 5 s. The fineness value of the ink was recorded.

#### 2.3.5. Thixotropic Properties of Inks

The thixotropic property test of inks was conducted in three steps. First, the shear rate was set to 0.1 s^−1^ and the test duration to 60 s. Next, the shear rate was set to 1000 s^−1^ and the test duration to 30 s. Finally, the shear rate was returned to 0.1 s^−1^ and tested for 300 s. These three steps corresponded to the ink’s structural state before, during, and after the printing process. The viscosity evolution was recorded throughout the test, along with the recovery extent and recovery time.

#### 2.3.6. Viscosity Properties of Inks

The viscosity was measured using a rotational rheometer (Kinexus Pro+, Malvern, UK). Test conditions were maintained at 23 °C and 50% RH. A shear rate of 0.1 s^−1^ was applied to measure the viscosity over a 300 s period.

#### 2.3.7. Flow Curve Properties of Inks

Using a rotational rheometer, the shear rate was increased from 0.1 s^−1^ to 1000 s^−1^ over 180 s and the variation in ink viscosity was recorded with shear rate.

### 2.4. Preparation and Characterization of Indicator Ink Labels

#### 2.4.1. Preparation

Ink-2 was selected as the freshness-indicating ink. Prior to printing, the ink was stirred for 1 h using a magnetic stirrer (HJ-6A, Shenzhen Dingxinyi Laboratory Equipment Co., Ltd., Shenzhen, China) to ensure uniform distribution, and air bubbles were removed from the ink using a vacuum drying oven (DZ-2BCII, Tianjin Tester Instrument Co., Ltd., Tianjin, China). The ink was screen-printed onto the surface of PLA film to form indicator labels or patterns. The prepared indicator ink was loaded onto a screen printing plate with pre-designed pattern. PLA film was fixed on a printing platform, and the ink was uniformly scraped onto the surface of the PLA film using a squeegee to form the uniform ink layer. After printing, the printed ink layer was dried naturally at room temperature for several minutes until the solvent was fully evaporated. The prepared labels were stored in a dry, dark environment at room temperature for later use.

The outline pattern shown in Figure 1 was only used to visually demonstrate the printing effect of the ink. For all performance tests including tensile, OTR, WVP and other characterizations, the PLA films were prepared with full ink coverage by screen printing. A 200-mesh screen was used for the printing process.

#### 2.4.2. Fourier Transform Infrared (FTIR)

Fourier transform infrared (FTIR) spectroscopy (Nicolet iS50, Thermo Fisher Scientific, Waltham, MA, USA) was used to analyze the chemical structure and interfacial interactions of the samples. The spectra were recorded in the wavenumber range of 4000–500 cm^−1^.

#### 2.4.3. Oxygen Transmission Rate (OTR) 

The oxygen transmission rate (OTR) was tested according to GB/T 1038.1-2022 [39] using the differential pressure gas permeameter (Labthink Classic 216, Labthink, Jinan, China). All tests were conducted under controlled conditions of 23 °C and 50% RH. Samples were cut into disks with a diameter of 97 mm and conditioned at 23 °C and 50% RH for 4 h before testing. Both the upper and lower chambers of the permeameter were degassed for 3 h before measurement. The oxygen transmission rate was then recorded.

#### 2.4.4. Water Vapor Permeability (WVP)

The water vapor permeability (WVP) was tested according to GB/T 1037-2021 [40] using a water vapor permeability tester (Labthink W3/060, Labthink, Jinan, China). The samples were cut into disks with a diameter of 97 mm and conditioned at 23 °C and 50% RH for 4 h. The conditioned sample was then placed in a moisture permeation cup. The test interval was set to 30 min. Measurements were conducted under test conditions of 38 °C and 90% RH.

#### 2.4.5. Mechanical Properties

For mechanical property testing, both the printed PLA film and the blank PLA film were cut into dimensions of 60 mm × 10 mm. The samples were conditioned at 23 °C and 50% RH for 4 h. Tensile strength and elongation at break were measured using an electronic tensile testing machine (3343, Instron, Norwood, MA, USA) at a test speed of 10 mm/min.

#### 2.4.6. Colorimetric Response to Ammonia Vapor

PLA films printed with the indicator labels were cut into 10 mm × 10 mm squares. Each square was attached to the inner side of a Petri dish lid. A specified volume of ammonium hydroxide solution was then placed into the corresponding petri dish to generate the required ammonia atmosphere. Tests were conducted at ammonia concentrations of 10 ppm, 20 ppm, and 50 ppm. Photographs were taken of the label condition at 0, 1, 3, 5, 10, 15, 30, and 60 min. And the color parameters (L, a, b) were measured using a portable colorimeter (TS10, 3NH Technology Co., Ltd., Shenzhen, China) with a D8 light source and a 10° observer angle. Before each measurement, the instrument was calibrated using a standard white and black plate, and the sample was placed tightly against the measurement aperture. The color difference value (ΔE) was calculated using Equation (1):

(1) $$∆E=\sqrt{{(L-L_{0})}^{2}+{(a-a_{0})}^{2}+{(b-b_{0})}^{2}}$$

In the formula, L_0_, a_0_, and b_0_ represent the initial lightness, red–green value, and yellow–blue value, respectively, while L, a, and b correspond to the lightness, red–green value, and yellow–blue value of the label after exposure to the ammonia atmosphere.

#### 2.4.7. Color Stability Under Storage Conditions

PLA films printed with the indicator labels were cut into 20 mm × 20 mm squares and stored under continuous light exposure in two different environments of 25 °C with 60% RH and refrigeration at 4 °C. At intervals of every five days, the samples were photographed, and their color parameters were tested.

#### 2.4.8. Reversibility of Color Change

PLA films printed with the indicator labels were attached to the inner side of Petri dish lids and allowed to equilibrate in the test environment for 2 h. 0.5 mL of 30% (*v*/*v*) ammonium hydroxide was added. After 5 min of exposure, the label was photographed, and its color parameters were measured using a spectrophotometer (TS10, 3nh, Guangzhou, China). Subsequently, the volatile ammonia was removed. Next, 0.5 mL of 30% (*v*/*v*) glacial acetic acid was added, and the same measurement procedure was followed. This cycle of alternately exposing the label to ammonia and acetic acid vapors was repeated 20 times. The test method was adapted from Ronte et al. [41].

### 2.5. Freshness-Indicating Application on Shrimp

Fresh shrimp were purchased from a local market. Shrimp of similar size were randomly selected and placed in glass bowls, with four shrimp per bowl. The bowls were sealed with PLA film printed with the curcumin-based indicator label. The bowls were then stored at 25 °C indoors and refrigerated at 4 °C for 48 h and 6 days, respectively. For samples stored at 25 °C, photographs of both the shrimp and the indicator label were taken every 8 h under consistent lighting and camera settings. The color parameters of the label were measured using a spectrophotometer, and the ΔE value was calculated. Concurrently, the TVB-N content and pH of the shrimp meat were analyzed. For samples stored at 4 °C, the same measurements were performed at daily intervals.

The TVB-N content was analyzed according to GB 5009.228-2016 [42] with slight modifications, using an automatic Kjeldahl nitrogen determinator (ZDDN-II, Top Cloud-Agri, Hangzhou, China). Shrimp meat was prepared by removing the head, shell, and tail, followed by homogenization. A total of 50 g of distilled water was added to 10 g of minced shrimp meat. The mixture was stirred using a magnetic stirrer for 30 min, then the mixture was allowed to stand, and the supernatant was collected. A total of 30 mL of supernatant, 15 mL of magnesium oxide suspension (10 mg/mL), and 30 mL of distilled water were added to the tube of the automatic Kjeldahl nitrogen determinator. The determination was initiated to obtain the sample distillate. A mixed indicator solution was prepared by combining methyl red ethanol solution (1 g/L) and bromocresol green ethanol solution (1 g/L) at a volume ratio of 1:5. For each titration, 30 mL of boric acid solution (20 g/L) was mixed with 20 μL of the mixed indicator to serve as the receiving solution. This solution was used to collect the distillate from the Kjeldahl apparatus, forming the test solution. The test solution was then titrated with a hydrochloric acid standard solution (0.01 mol/L), and the volume of the titrant consumed was recorded. The TVB-N content was calculated using Equation (2):

(2) $$\mathrm{TVB}-N=\frac{(V_{1}-V_{2})\times c\times 14}{m\times (V/V_{0})}\times 100$$

In Equation (2), TVB-N represents the total volatile basic nitrogen content of the sample, expressed in mg/100 g. V_1_ is the volume of the standard titration solution consumed by the sample test solution (mL). V_2_ is the volume of the standard titration solution consumed by the blank test solution (mL). The c denotes the concentration of the hydrochloric acid standard solution (mol/L). The m is the mass of the shrimp meat sample (g). V is the volume of the supernatant filtrate taken for analysis (mL), and V_0_ is the total volume of the supernatant (mL). The value 14 corresponds to the nitrogen equivalent (g/mol) per milliliter of the hydrochloric acid standard titration solution consumed, and 100 is the unit conversion factor.

The pH was analyzed according to GB 5009.228-2016 with minor modifications. A total of 27 g of distilled water was mixed with 3 g of minced shrimp meat. The mixture was stirred using a magnetic stirrer for 30 min and then allowed to stand. The pH of the resulting supernatant was measured with a digital pH meter.

### 2.6. Statistical Analysis

The data presented are the mean of three independent measurements performed under the same experimental conditions, and the error bars represent the relative standard deviation (RSD). Statistical analysis and graph plotting were performed using Origin 2021 software.

## 3. Results and Discussion

### 3.1. Particle Size and Zeta Potential of Inks

**Figure 2:**
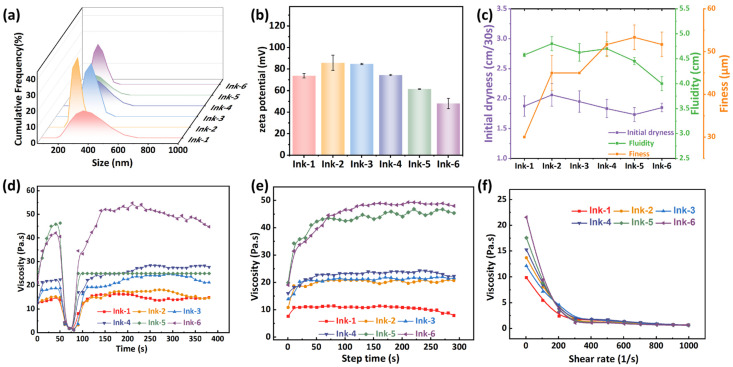
Characterization of inks: (**a**) Particle size. (**b**) Zeta potential. (**c**) Initial dryness, fluidity, and fineness. (**d**) Thixotropic. (**e**) Viscosity. (**f**) Flow curve.

The particle size distribution and zeta potential of the inks are shown in Figure 2a and Figure 2b, respectively. Among them, Ink-2 exhibits a unimodal distribution with the smallest particle size distribution area, indicating the most uniform particle dispersion within the ink. The smallest particle size of Ink-2 is beneficial for achieving better printing performance [43]. The zeta potential value is commonly used to assess the stability of a dispersion system. A higher absolute zeta potential value indicates stronger electrostatic repulsion between particles, which helps prevent particle aggregation and results in a more stable system. Generally, a system is considered highly stable when the absolute zeta potential exceeds 30 mV [44]. As shown in Figure 2b, except for Ink-6, all inks exhibit zeta potentials greater than 50 mV, with Ink-2 showing the highest value of 85.8 mV. EC and PVB contain hydroxyl groups that can impart weak negative charges when adsorbed onto particle surfaces. In Ink-2, the two polymers likely form a compact and well-organized adsorption layer, maximizing the surface charge density and thus the zeta potential [45,46].

### 3.2. Initial Dryness, Fluidity, and Fineness of Inks

Figure 2c illustrates the printability of the six inks with varying compositions. Regarding the initial dryness of the inks, as the concentration of PVB decreased and EC increased, the drying rate initially rose and then declined. This trend may be attributed to the stronger affinity of PVB for ethanol compared to EC. PVB contains abundant hydroxyl groups along its molecular chain, which can form strong hydrogen bonds with ethanol molecules, thereby retarding solvent evaporation. In contrast, EC has fewer free hydroxyl groups due to the substitution by ethoxyl groups, resulting in weaker interaction with ethanol and faster drying. As the PVB concentration decreased, the fluidity of the inks showed a decreasing trend, which is inversely proportional to their viscosity. Fineness reflects the degree of dispersion of solid fillers within the binder. A smaller fineness leads to superior printing performance, resulting in more uniform color distribution, sharper pattern definition, and higher gloss [45]. The data in Figure 2c indicate that ink fineness data increases with decreasing PVB concentration, indicating more pronounced agglomeration of fillers and poorer dispersion. As reported by Li et al. [47], the degree of neutralization affects the solubility of particles in the solvent, which in turn influences the fineness of the system. Therefore, when PVB is the dominant component in the binder, the compatibility of the overall system with ethanol reaches an optimum, allowing curcumin particles and any trace aggregates to be fully dispersed. Overall, Ink-2 should be selected as the indicator ink due to its slightly slower drying rate, moderate fluidity, and low fineness.

### 3.3. Thixotropic, Viscosity, and Flow Curve Properties of Inks

Thixotropy reflects the structural destruction and recovery ability of inks under shearing, which directly affects the ink transfer performance during printing and the pattern setting effect after printing [48]. As shown in Figure 2d, all inks exhibit typical thixotropic behavior. During the high-shear stage, the ink viscosity drops sharply to below 1 mPa·s, and the entangled polymer network is destroyed, which facilitates the ink passing through the screen plate [49]. Except for Ink-6, all inks can recover to their initial state. Moreover, the viscosity of the recovered inks remains stable, indicating that the ink system is well-formulated and stable [50]. The incomplete recovery of Ink-6 may be attributed to its high EC proportion. Compared with PVB, EC has poorer solubility and compatibility with ethanol, and weaker intermolecular hydrogen-bonding interactions. In the EC-dominated system of Ink-6, a stable three-dimensional entanglement network cannot be formed, making it difficult to reconstruct the network structure after high shear, thus resulting in poor thixotropic recovery. As shown in Figure 2e, the viscosity of the inks decreases rapidly with increasing shear rate, exhibiting a shear-thinning behavior characteristic of non-Newtonian fluids. This reduction in viscosity can be attributed to the alignment of polymer molecules along the flow direction under shear stress, which weakens intermolecular interactions and reduces chain entanglements, thereby lowering flow resistance [51]. Figure 2f shows that the shear viscosity of the inks increased as the PVB concentration decreased. All viscosities are greater than 10 Pa·s, which is suitable for screen printing.

### 3.4. Characterization of Indicator Ink Labels

#### 3.4.1. FTIR

**Figure 3:**
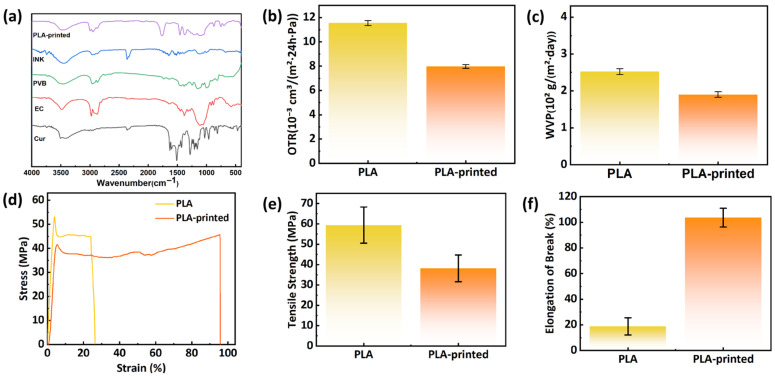
Characterization of original PLA film and PLA-printed film. (**a**) FTOR. (**b**) OTR. (**c**) WVP. (**d**) Stress–strain curve. (**e**) Tensile strength. (**f**) Elongation at break.

Figure 3a shows the FTIR spectra of Cur, EC, PVB, the formulated ink, and the screen-printed PLA film (PLA-printed). Curcumin shows a characteristic phenolic –OH stretching peak at around 3507 cm^−1^. The strong absorption bands at 1627 cm^−1^ and 1510 cm^−1^ are associated with the conjugated C=O and C=C structures, respectively. Additional characteristic peaks corresponding to aromatic ring stretching, olefinic C–H in-plane bending, and C–O stretching vibrations are also detected at 1602 cm^−1^, 1429 cm^−1^, and 1282 cm^−1^ [52]. For PVB, the absorption peaks at 2967 cm^−1^, 2942 cm^−1^, and 2925 cm^−1^ are assigned to the stretching vibrations of –CH_3_, –CH_2_, and –CH groups, respectively [53]. The band at 1730 cm^−1^ arises from carbonyl stretching, while the peaks at 1106 cm^−1^ and 968 cm^−1^ are characteristic of acetal groups. In the spectrum of EC, the peak near 2900 cm^−1^ corresponds to C–H stretching, and the absorption at 1154 cm^−1^ is attributed to the stretching vibration of the C–O–C ether linkage [54]. The characteristic absorption peaks of PVB, EC, and curcumin can still be observed in the ink spectrum. Notably, the –OH absorption band becomes broader and slightly shifts to lower wavenumbers, suggesting the formation of hydrogen bonding interactions among the ink components. These results confirm that the indicator ink was successfully immobilized onto the PLA film through physical interactions.

#### 3.4.2. OTR

During food storage, nutrients in the food can react with oxygen, leading to a decline in nutritional value and freshness. Therefore, packaging with a low OTR can effectively extend the shelf life and safety of food products [55]. As shown in Figure 3c, after printing with the ink, the OTR of the PLA film decreases from 11.55 × 10^−3^ cm^3^/(m^2^·24 h·Pa) to 7.96 × 10^−3^ cm^3^/(m^2^·24 h·Pa). The reduction is likely attributed to the rigid molecular chains and dense packing of EC in the ink, which enhances the oxygen barrier. The excellent adhesion and interface between the ink and the PLA film substrate result in a denser film structure, thereby reducing pathways for oxygen permeation.

#### 3.4.3. WVP

The moisture barrier property of packaging is a crucial factor affecting the shelf life of many food products. So it serves as a key parameter for evaluating the suitability of packaging materials. For food packaging, a lower water vapor transmission rate reduces moisture evaporation from food, inhibits microbial growth, and thereby maintains food freshness [56]. As shown in Figure 3d, the WVP of the pristine PLA film is 2.52 × 10^2^ g/(m^2^·day). After printing, the WVP decreased to 1.90 × 10^2^ g/(m^2^·day). This result is likely because the printed ink layer fills surface imperfections and elongates the diffusion pathway for water vapor, making it more difficult for moisture to permeate through the film.

#### 3.4.4. Mechanical Properties

As shown in Figure 3e, after printing, the tensile strength of the film decreased slightly, while its elongation at break increased significantly. Similar mechanical changes have been reported in printed polymer films [57], which were attributed to the formation of a flexible surface coating. The printed ink layer forms a continuous, relatively low modulus coating on the PLA surface. The improved elongation at break mainly originates from the good flexibility and ductility of the ink layer. During tensile deformation, the surface coating can effectively redistribute stress and reduce stress concentration at defects on the PLA surface, thereby enhancing the ductility of the composite film. Food packaging film should possess a certain degree of flexibility. Although printing slightly reduces the strength of the film, the improved toughness makes it more suitable for packaging items of various sizes and shapes [58].

### 3.5. Ammonia Response, Color Stability, and Reversibility of Labels

**Figure 4:**
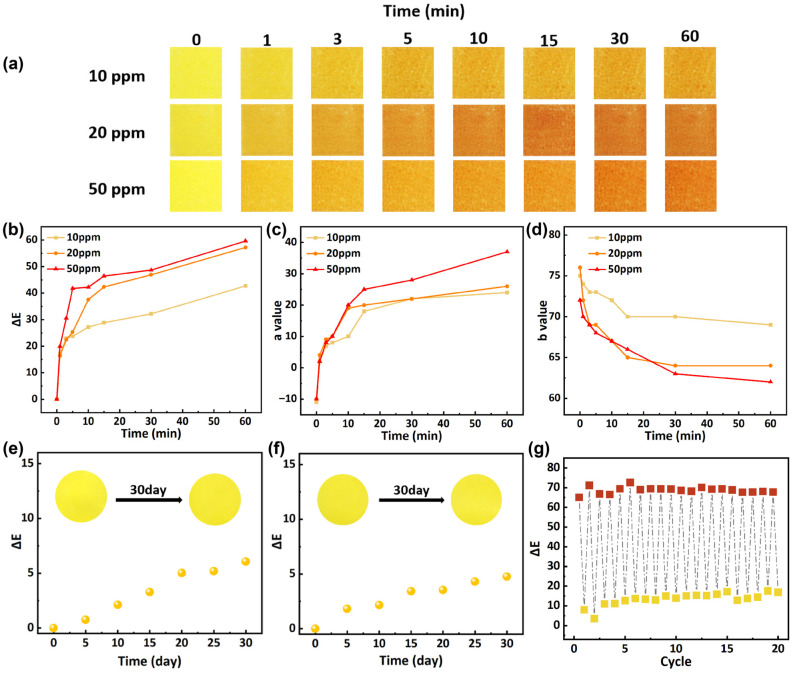
(**a**) Ammonia response of indicator ink labels. (**b**) Changes in the color parameter ΔE, (**c**) color parameter a, and (**d**) color parameter b during the exposure process. (**e**) Color change in labels stored at 25 °C. (**f**) Color change in labels stored at 4 °C. (**g**) Reversibility of ΔE values for labels under successive exposure to ammonia and acetic acid vapors.

Figure 4a depicts the colorimetric response of the printed PLA film to ammonia at concentrations of 10 ppm, 20 ppm, and 50 ppm. When exposed to ammonia at concentrations of either 50 ppm or 20 ppm, the label undergoes a rapid color transition from yellow to deep reddish-brown. Even at 10 ppm, the film still exhibited a detectable color change after a period of exposure, indicating its potential for monitoring early-stage spoilage. As shown in Figure 4c,d, an increase in the a value and the decrease in the b value indicate a color shift toward red and yellow, respectively. Figure 4b shows that the ΔE value increased sharply within the first minute and continued to rise over time, with the rate of increase slowing after 10 min. At all three ammonia concentrations, the ΔE value exceeded 20 by the 3 min mark, representing a change clearly visible to the naked eye. This high sensitivity to ammonia enables the system to effectively monitor ammonia dynamics within the packaging, supporting real-time tracking of food spoilage progression.

Curcumin exhibits limited stability and is susceptible to degradation under environmental stressors such as light and temperature variations [59]. To validate reliability under actual food distribution and storage conditions, the color stability of the printed PLA film was evaluated over 30 days under simulated refrigeration (4 °C) and ambient (25 °C, 50% RH) environments, as shown in Figure 4e,f. Throughout the 30-day observation period, all samples exhibited no discoloration, fading, or significant physical deformation. Under ambient conditions, the ΔE value increased slightly to 6.05, while under refrigerated storage at 4 °C, it increased to 4.75. Jeovan A. Araujo et al. [60] exposed curcumin-incorporated PLA films to UV light under both dry and condensing conditions to simulate sunlight-induced degradation. A noticeable color change was observed on day 3, with a color difference (ΔE) value reaching 4.8. In comparison, the film demonstrated better color stability under refrigerated conditions. Although a slight color drift was observed, the change in ΔE was significantly smaller than the ΔE variation observed during the film’s ammonia response. Therefore, the film exhibits excellent color stability for food packaging applications, particularly in cold chain systems, with minimal indication error.

To evaluate practical durability, the printed PLA film was subjected to 20 cycles of reversibility testing by alternating exposure to ammonia and acetic acid vapors. This procedure simulates the repeated color changes the material might undergo during actual use due to spoilage volatiles and environmental fluctuations. As shown in Figure 4g, upon exposure to ammonia, the color rapidly changed from the original yellow to a distinct reddish-brown. When subsequently exposed to acetic acid vapor, the color reverted completely to its original yellow. The color change in curcumin under acidic and alkaline vapors is based on its reversible structural transformation. Under neutral or acidic conditions, curcumin exists predominantly in the enol form and exhibits a bright yellow color. When exposed to ammonia vapor, r, curcumin undergoes deprotonation to form a conjugated enolate anion. This transformation extends the conjugated system and causes a red shift in the absorption spectrum, leading to orange-red color. The reaction is reversible because the transformation involves only proton transfer without breaking the covalent structure of curcumin. Throughout the 20 test cycles, the ΔE values remained largely stable. Arnat Ronte et al. [41] observed the reversibility of dye color responses using the same method and obtained similar results. These results demonstrate its long-term functional reliability, indicating its capability for reliably monitoring the freshness of high-protein foods, such as shrimp, during extended storage and transportation.

### 3.6. Application for Shrimp Freshness Indicating

**Figure 5:**
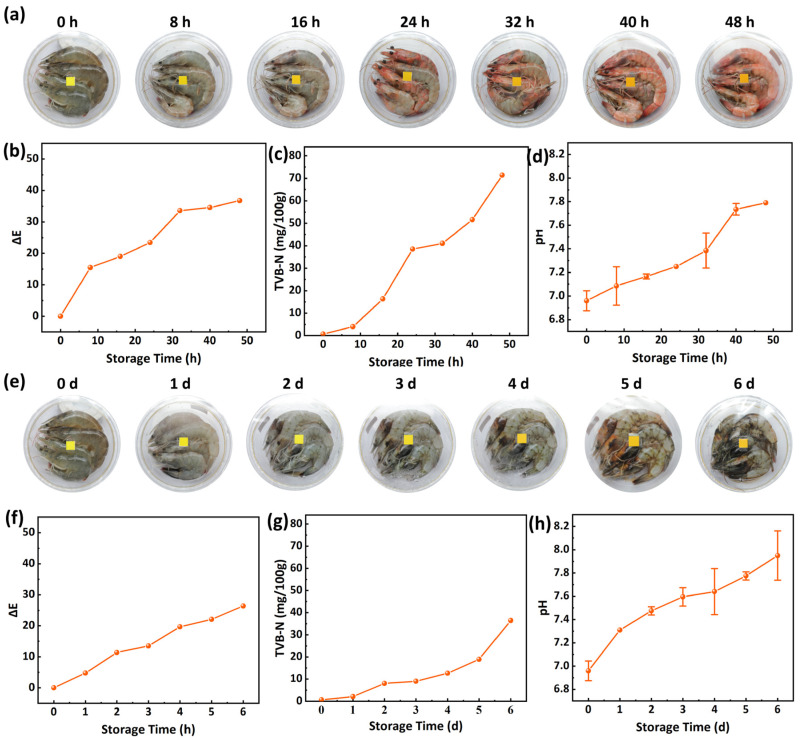
(**a**) Photographs of shrimp freshness monitoring at 25 °C. (**b**) Changes in ΔE values of labels in shrimp freshness monitoring at 25 °C. (**c**) Changes in TVB-N of shrimp stored at 25 °C. (**d**) Changes in pH of shrimp stored at 25 °C. (**e**) Photographs of shrimp freshness monitoring at 4 °C. (**f**) Changes in ΔE values of labels in shrimp freshness monitoring at 4 °C. (**g**) Changes in TVB-N of shrimp stored at 4 °C. (**h**) Changes in pH of shrimp stored at 4 °C.

Figure 5 illustrates the application of the freshness-indicating packaging for the storage of shrimp. The correlation between the color change in the indicator label of the packaging and shrimp spoilage was established by measuring the TVB-N content and pH level [61]. During storage at 25 °C, the appearance of the fresh shrimp gradually changed from a pale bluish-green to red as spoilage progressed (Figure 5a). This color change is a natural phenomenon resulting from the degradation of the protein–astaxanthin complex in shrimp during spoilage. A distinct color change in the indicator zone of the packaging was visibly detectable at 16 h, transitioning from the initial bright yellow to an orange-yellow, with a corresponding ΔE value of 19. The ΔE value greater than 5 is considered clearly visible to the naked eye (Figure 5b). At this point, the shrimp exhibited a slight reddening. In the meantime, the TVB-N value reached 16.3 mg/100 g, indicating a state of slight spoilage. By 24 h, the indicator label had turned orange, the shrimp showed significant reddening, and the TVB-N value reached 38.4 mg/100 g, signifying complete spoilage of the shrimp meat (Figure 5c), in accordance with the limit specified in GB 2733-2015 [62]. Throughout the storage period, the pH of the shrimp meat gradually increased. It is likely due to the accumulation of nitrogenous compounds and the release of amines resulting from microbial growth, which aligns with the TVB-N results [63]. The freshness-indicating packaging demonstrated a color change even when visual changes in the shrimp were subtle, confirming its high sensitivity to the spoilage progression of shrimp and its effectiveness as a freshness indicator.

Under simulated cold storage conditions at 4 °C, the spoilage progression of shrimp was significantly delayed. And the indicator label of the packaging correspondingly displayed a different dynamic. As shown in Figure 5e, during the first three days of storage, the appearance of the shrimp showed no obvious change. The color of the indicator label remained stable with no visually detectable alteration. The ΔE value fluctuated within a low range. On day 3, a distinct color transition began. The ΔE value reached 13.5, which is easily noticeable. It continued to increase on days 5 and 6 (Figure 5f). As spoilage progressed, the shrimp’s color gradually darkened from pale bluish-green. The head began to darken on day 3, and clear spoilage was visible by day 5. The TVB-N value accumulated slowly in the first four days. It reached 9.9 mg/100 g on day 3, indicating slight spoilage. By day 5, the TVB-N value reached 19.9 mg/100 g, marking the stage of clear spoilage (Figure 5g). The change in pH followed a similar upward trend (Figure 5h). The clear color change in the label on day 3 matched the time when the TVB-N value passed the food safety limit. This shows that in extended cold chain storage, the indicator packaging remains effective at low temperatures. It demonstrates potential for visual freshness monitoring across different storage and transport conditions.

## 4. Conclusions

This study successfully developed a curcumin-based freshness-indicating ink and integrated it onto biodegradable PLA film via screen printing to construct a structurally integrated intelligent packaging system. The formulated ink exhibited excellent printability and optimal thixotropic properties, enabling the fabrication of uniform, clear, and stable indicator labels on PLA substrates. This label is integrally molded with the PLA film rather than using traditional adhesive methods, offering enhanced stability and a simpler, more convenient manufacturing process. The uniformly formed ink indicator label layer enhances the oxygen and moisture barrier properties of the packaging film while maintaining its flexibility. The indicator label demonstrated high sensitivity and a rapid, visible colorimetric response to ammonia, correlating closely with the spoilage progression of shrimp during storage at both 25 °C and 4 °C. This work provides a practical, low-cost, and environmentally friendly strategy for real-time visual monitoring of food freshness. Although shrimp was employed as the model food in this study, the developed indicator shows great potential for further extension to other protein-rich foods, such as fish, pork, and beef, which also generate alkaline volatile components during spoilage. It shows significant potential to reduce food waste and improve supply chain transparency for perishable products.

## Figures and Tables

**Table 1:** Component content of each ink.

Name	EC/g	PVB/g	Curcumin/g
Ink-1	0.1	0.9	0.2
Ink-2	0.2	0.8	0.2
Ink-3	0.3	0.7	0.2
Ink-4	0.4	0.6	0.2
Ink-5	0.5	0.5	0.2
Ink-6	0.6	0.6	0.2

## Data Availability

The original contributions presented in this study are included in the article. Further inquiries can be directed to the corresponding author.

## Abbreviations

The following abbreviations are used in this manuscript:

PLA	polylactic acid
EC	ethyl cellulose
PEG 400	polyethylene glycol 400
PVB	polyvinyl butyral
TVB-N	total volatile basic nitrogen
OTR	oxygen transmission rate
WVP	water vapor permeability
ΔE	color difference value
